# A Cross-Sectional Study of Rift Valley Fever Exposure in Humans and Livestock in Southwestern Uganda Using a One Health Approach: Evidence of Elevated Seroprevalence Outside Recognized Outbreak Periods

**DOI:** 10.3390/pathogens15020224

**Published:** 2026-02-17

**Authors:** Luke Nyakarahuka, Silvia Situma, Raymond Odinoh, Barnabas Bakamutumaho, Carolyne Nasimiyu, Jeanette Dawa, Justine Okello, Honest Kemiyondo, Alex Tumusiime, Mutesi Joanita, Jackson Kyondo, John Kayiwa, David Odongo, Deo Birungi Ndumu, Kariuki M. Njenga, Robert F. Breiman

**Affiliations:** 1 Uganda Virus Research Institute, Entebbe P.O. Box 49, Uganda; bbarnabas2001@yahoo.com (B.B.); justinokello01@gmail.com (J.O.); atumusiimeug@gmail.com (A.T.); mutesijoanita4@gmail.com (M.J.); jacksonkyondo@gmail.com (J.K.); jkayiwa@yahoo.com (J.K.); davidodongojr@gmail.com (D.O.); 2 Rollins School of Public Health, Emory University, 1518 Clifton Rd NE., Atlanta, GA 30322, USA; rfbreiman@emory.edu; 3 Department of Biosecurity, Ecosystems and Veterinary Public Health, College of Veterinary Medicine, Animal Resources and Biosecurity, Makerere University, Kampala P.O. Box 7062, Uganda; 4 Center for Research in Emerging Infectious Diseases East and Central Africa, Nairobi 00202, Kenya; silvia.situma@wsu.edu (S.S.); carolyne.nasimiyu@wsu.edu (C.N.); jdawa@cartafrica.org (J.D.); mkariuki.njenga@wsu.edu (K.M.N.); 5 Department of Animal Science, Pwani University, Kilifi 80108, Kenya; 6 Washington State University Global Health Program-Kenya, Nairobi 00200, Kenya; 7 Department of Animal Health and Production, Ministry of Agriculture, Animal Industry and Fisheries, Entebbe P.O. Box 513, Uganda; khonestie@gmail.com (H.K.); ndumudb@gmail.com (D.B.N.); 8 Paul G. Allen School for Global Health, Washington State University, Pullman, WA 98165, USA; 9 Infectious Diseases and Oncology Research Institute, University of the Witwatersrand, Johannesburg 2000, South Africa

**Keywords:** rift valley fever, seroprevalence, one health, zoonotic diseases, livestock, cattle, Uganda, vector-borne diseases, epidemiology, risk factors

## Abstract

Rift Valley fever (RVF) transmission has intensified in southwestern Uganda since 2016. To quantify human and livestock exposure and associated risks, we conducted a cross-sectional serosurvey in Isingiro, Kabale and Rubanda districts between October and November 2023. A total of 766 humans and 2383 livestock were sampled and tested for RVF antibodies using ELISA, with structured questionnaires capturing demographic, behavioral and environmental data. Human seroprevalence was 11.5% (88/766), varying by district (13.8% Isingiro, 11.8% Rubanda, 6.8% Kabale; *p* = 0.04). Independent predictors from the multivariate model included raw-meat consumption (aOR 6.11; 95% CI 1.16–27.80), cattle ownership (aOR 2.33; 95% CI 1.27–4.36), male sex (aOR 1.64; 95% CI 1.02–2.66) and younger age compared with ≥50 years (31–49 years: aOR 2.02; 95% CI 1.20–3.48; 18–30 years: aOR 2.37; 95% CI 1.04–5.14). Herd-level seroprevalence was 42.5% (204/480), associated with cattle presence (aOR 6.48; 95% CI 4.10–10.40), lack of carcass burial (aOR 15.70; 95% CI 4.23–63.60), on-farm slaughter (aOR 2.14; 95% CI 1.21–3.89) and increased mosquito activity (aOR 1.75; 95% CI 1.13–2.73). Animal-level seroprevalence was 14.6% (347/2383), highest in cattle (33.8%), with cattle having markedly higher odds than goats (aOR 6.73; 95% CI 4.96–9.14). These findings demonstrate substantial transmission and highlight cattle-centered interfaces as primary targets for control to humans.

## 1. Introduction

Rift Valley fever (RVF), a zoonosis primarily affecting livestock, particularly cattle, sheep, and goats, can spill over into humans, a proportion of whom develop hemorrhagic fever with a potential for mortality. First described in Africa in the 1930s, RVF has since been responsible for multiple outbreaks in both humans and animals across East Africa, the broader African continent, and the Arabian Peninsula [1,2,3]. RVF outbreaks have significant economic implications, disrupting trade and livestock production in affected regions. For instance, the 2000 RVF outbreak in Saudi Arabia and Yemen led to widespread bans on livestock exports from the Horn of Africa, severely impacting regional economies [4].

While *Aedes* and *Culex* mosquitoes are the primary vectors for RVF transmission in livestock, interactions between vectors, flooding events, livestock handling practices and mobility and the proximity of humans contribute to a complex epidemiology for sporadically occurring and epidemic disease in humans and animals [5]. Outbreaks are often linked to periods of heavy rainfall and flooding, which create ideal breeding conditions for mosquito vectors. While vaccines for livestock exist, their efficacy is limited, they are not widely used, and no approved human vaccine is currently available, leaving both animal and human populations vulnerable [6,7]. The epidemiology of RVF provides a clear rationale for a highly functional, integrated, One Health approach for public health disease control and prevention.

Rift Valley fever re-emerged in Kabale District in 2016 following nearly 50 years of sporadic, rarely diagnosed disease, with laboratory confirmation of infections in both humans and livestock [7]. This outbreak highlighted the interconnectedness of human and animal health, with studies indicating similar seroprevalence in humans (12%) and livestock (13%). Among the humans, butchers and others who handle raw meat had the highest identified risk for infection, while young animals had the lowest risk for infection [8]. Recent RVF outbreaks in central and southwestern Uganda (2017–2020) and national serosurveys have revealed significant seropositivity in livestock of up to 22% in high-risk areas correlated with ecological factors such as wetlands and flooding [9,10]. Geostatistical models further identified spatial risk patterns driven by climate and environment [11,12]. These findings underscore the need for updated seroepidemiological data to guide surveillance, vaccination, and disease prevention efforts.

Southwestern Uganda represents a distinctive ecological and epidemiological setting for Rift Valley fever (RVF) transmission. RVF was first detected in this region in 2016 in Kabale District, followed by recurrent outbreaks and evidence of sustained transmission. A linked health facility-based surveillance study conducted in the same region demonstrated elevated RVF seroprevalence outside recognized outbreak periods, providing the impetus for the present community-based investigation [13]. The region is characterized by high cattle density, mixed crop–livestock production systems, favorable climatic conditions for mosquito survival, and substantial livestock movement across borders with Tanzania, Rwanda, and the Democratic Republic of Congo. These features make southwestern Uganda an appropriate setting to examine RVF transmission dynamics at the human–animal–environment interface.

We designed and implemented the Seroprevalence and Ecologic factors Associated with RVF (SPEAR) study to evaluate the burden of RVF on both animal and human populations in Kabale, Rubanda, and Isingiro districts in southwestern Uganda and to assess risk factors associated with RVF seropositivity using an integrated, human and Veterinary health (One Health) approach. The One Health aproach was operationalized through household-linked sampling of humans and their domestic livestock, with concurrent serological testing conducted using standardized laboratory protocols. Data were collected simultaneously by an interdisciplinary team of public health and veterinary scientists, and integrated analyses were performed to evaluate shared and species-specific risk factors for RVF exposure. We sought to fill critical gaps in knowledge of the epidemiology and transmission dynamics of RVF in Uganda for use in optimizing public health prevention and control strategies and to set future research priorities.

## 2. Materials and Methods

### 2.1. Study Design and Setting

**Figure 1:**
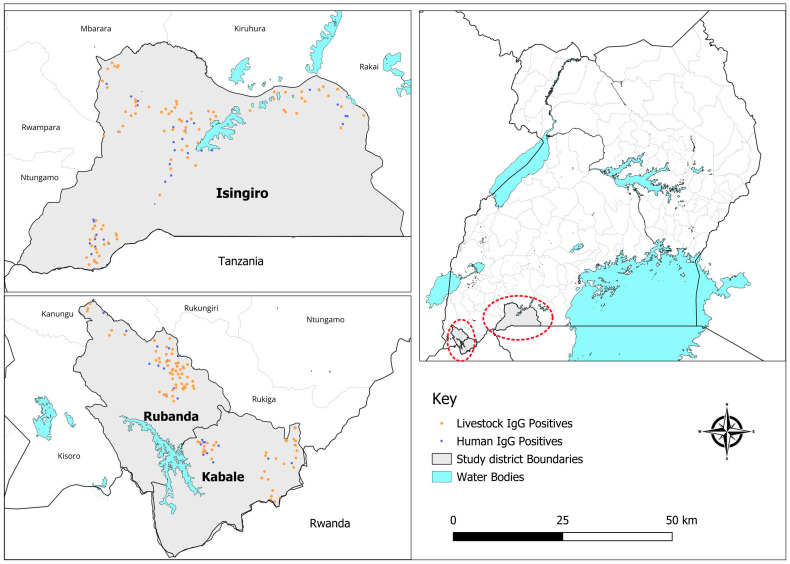
Human and animal seropositivity and sampling sites across Kabale, Rubanda, and Isingiro.

This was a cross-sectional study conducted from October 30th to November 25th, 2023. The research was carried out in three districts of Kabale, Rubanda and Isingiro located in southwestern Uganda. Kabale and Rubanda districts, formerly a single administrative unit, feature a mix of volcanic hills and valleys with a rainforest ecosystem in the west; the area borders Rwanda to the east. Isingiro District, bordering Tanzania, is characterized by rangelands and volcanic hills with valleys (Figure 1). The economic activities in all three districts are focused on crop farming and livestock keeping.

### 2.2. Study Population and Sampling

The study population included humans aged ≥1 year and livestock (cattle, sheep, and goats) from randomly selected households across the three districts. One human participant per household was randomly selected for enrollment, while livestock sampling included up to five cattle, five sheep, and five goats per household. Sample size determination was based on prevalence estimates from prior studies, which indicated RVF seropositivity rates of 12% in humans, 27% in cattle, 7% in goats, and 4% in sheep [8]. Considering these estimates and a 10% non-response rate, the target sample sizes were calculated as 765 humans and 2476 livestock (982 cattle, 813 goats, and 681 sheep) across the three districts. Assuming a significance level of 0.05 and 80% power, our study was powered to detect a minimum risk ratio of approximately 1.5 for livestock seroprevalence comparisons and 2.0 for human seroprevalence, depending on the exposure variable. Random geocodes were used to identify 765 households for enrollment, including livestock keeping and non-livestock keeping with additional replacement geocodes prepared to address non-response or inaccessibility as previously done in a related study in Kenya [14]. Sub-counties were randomly selected to ensure representative sampling, and sample size allocation within sub-counties was proportional to population distribution.

### 2.3. Data Collection

Three structured questionnaires were administered to collect comprehensive data at different levels. The first questionnaire targeted the household level, focusing on known or potential risk factors for RVF such as geographic location, household practices, and environmental exposures. The second questionnaire was administered to a randomly selected household participant to gather individual-level data, including socio-demographics, behaviors and knowledge including biosecurity practices, animal contact types, consumption of animal products, and RVF awareness. The third questionnaire focused on sampled livestock, documenting individual animal-level risk factors such as sex, age, breed, production system, vaccination history, abortion history, herd management practices and other factors. This multi-tiered approach allowed for a holistic assessment of RVF risk factors across human and animal populations. Blood samples were collected from all human participants and selected livestock. For humans, a 4 mL venous blood sample was obtained using standard aseptic techniques, while 6 mL venous blood samples were collected from livestock using appropriate restraint to ensure humane handling.

### 2.4. Laboratory Analysis

Blood samples were transported and maintained under cold chain conditions (2–8 °C) to the Uganda Virus Research Institute (UVRI) for analysis. The presence of total RVF antibodies in both human and livestock samples was determined using a commercially available multi-species competitive enzyme-linked immunosorbent assay (C-ELISA) from IDvet^®^ (Grabels, France), performed strictly according to the manufacturer’s instructions. The assay’s validity was confirmed if the mean OD of the positive control (ODPC) was less than 30% of the negative control’s mean OD (ODNC), i.e., ODPC/ODNC < 0.3, and if the ODNC was greater than 0.7 (ODNC > 0.7). All samples were tested in duplicate, and indeterminate results were classified as negative. The diagnostic performance of the IDvet kit includes a reported sensitivity of 98% and specificity of 100%.

### 2.5. Data Analysis

Data were analyzed using R statistical software (Version 2023.06.0). Descriptive statistics were used to summarize the demographic, clinical, and environmental characteristics of study participants and animals and household level. Continuous variables were reported as medians with interquartile ranges (IQRs), while categorical variables were presented as frequencies and percentages. Comparisons between groups (e.g., seropositive vs. seronegative) were performed using appropriate statistical tests: Pearson’s chi-squared test or Fisher’s exact test for categorical variables and the Wilcoxon rank-sum test for continuous variables. Bivariate analysis was conducted to identify associations between independent variables (e.g., demographic characteristics, livestock management practices, and environmental factors) and RVF seropositivity. Variables with a *p*-value ≤ 0.2 in bivariate analysis were considered for inclusion in the multivariate logistic regression model. A multivariable logistic regression was used to identify independent predictors of RVF seropositivity. Covariates were selected a priori based on epidemiological relevance and the prior literature, and district was retained in all models. Associations are reported as adjusted odds ratios (aORs) with 95% confidence intervals (CIs). All tests were two-sided with α = 0.05.

### 2.6. Ethical Considerations

This study was approved by the Uganda Virus Research Institute Research Ethics Committee (GC/127/849) and the Uganda National Council for Science and Technology (HS1713ES). Administrative clearance was obtained from the Ministry of Health. Informed consent was provided by all human participants or their guardians, and assent was obtained for minors. Consent for sampling livestock was obtained from the household head or animal owner. All procedures involving animals adhered to ethical standards for humane handling and sample collection.

## 3. Results

### 3.1. Human Demographic Summary

We enrolled 766 households/participants across three districts: Isingiro 383 (50.0%), Kabale 205 (26.8%), and Rubanda 178 (23.2%). The median household size was 4 (IQR: 3–5); the median age of participants was 39 years (IQR: 27–54). Other demographic characteristics of the participants, including gender, occupation, and socio-economic status, are provided in Table 1.

### 3.2. Human RVF Seroprevalence Across Demographic Categories

Overall human RVF seroprevalence was 11.5% (88/766), with significant variation by district (Isingiro 13.8%, Rubanda 11.8%, Kabale 6.8%; *p* = 0.04; Table 1, Figure 1). At sub-county level, seroprevalence varied from 6.9% (Birere) to 18.0% (Isingiro Town Council); these comparisons were not statistically significant after stratification. Seropositivity increased with age (*p* < 0.001), from 0% among those 0–17 years (n = 64) to 7.2% in 18–30 years, 10.6% in 31–49 years, and 18.5% in ≥50 years. Males had higher seropositivity than females (13.6% vs. 9.8%; *p* = 0.10). By occupation, seropositivity was highest among those reporting animal husbandry (25.5%) and lowest among students (1.5%) (overall *p* = 0.002).

### 3.3. Assessing Risk Factors for Human Disease

In unadjusted comparisons (Supplementary Table S1), RVF seropositivity was higher among individuals from cattle-keeping households, among those using rainwater or natural sources rather than piped water, and among those reporting livestock contact; most other variables were not associated. In adjusted models (Table 2), residence in Isingiro, male sex, cattle ownership (aOR 2.33, 95% CI 1.27–4.36); younger age, compared with ≥50 years, was associated with higher odds of RVF seropositivity (31–49 years: aOR 2.02, 95% CI 1.20–3.48; 18–30 years: aOR 2.37, 95% CI 1.04–5.14). Other risk factors identified included raw-meat consumption (aOR 6.11, 95% CI 1.16–27.80) and current illness (aOR 4.45, 95% CI 1.66–11.30), whereas increases in mosquitoes and RVF awareness were not significantly associated with RVF seropositivity.

### 3.4. Seroprevalence of RVF at Herd-Level

Herd-level RVF seroprevalence (≥1 seropositive animal per herd) was 42.5% (204/480 herds). In the multivariable model (Table 3), herds that included cattle had markedly higher odds of seropositivity (aOR 6.48, 95% CI 4.10–10.40). Herd size was inversely associated with seropositivity relative to large herds (medium: aOR 0.39, 95% CI 0.19–0.79; small: aOR 0.23, 95% CI 0.11–0.46). Carcass disposal methods showed strong effects compared with burial with soil cover: no burial (aOR 15.70, 95% CI 4.23–63.60) and on-farm slaughter without safe disposal (aOR 2.14, 95% CI 1.21–3.89) were associated with higher odds; other methods were not significant (aOR 1.10, 95% CI 0.48–2.49). Reported increases in mosquito activity were associated with higher odds (aOR 1.75, 95% CI 1.13–2.73). District differences were not statistically significant overall (Kabale vs. Isingiro: aOR 0.72, 95% CI 0.40–1.28; Rubanda vs. Isingiro: aOR 1.39, 95% CI 0.79–2.45; *p* = 0.10).

### 3.5. Animal-Level Livestock RVF Seroprevalence

We sampled 2383 animals: 681 cattle (29%), 1170 goats (49%), and 532 sheep (22%). Most were indigenous breeds (87%, 2073/2383), with crossbreeds 12% (283/2383) and exotics 1.1% (27/2383); females 85% (2031/2383). Overall total antibody seroprevalence was 14.6% (347/2383) and differed by species—cattle 33.8%, sheep 7.3%, goats 6.7% (*p* < 0.001). In adjusted models (Table 4), cattle had markedly higher odds of seropositivity than goats (aOR 5.83, 95% CI 4.20–8.07; *p* < 0.001), whereas sheep did not differ from goats (aOR 1.08, 95% CI 0.72–1.62; *p* = 0.711). Illness history was independently associated with higher odds (aOR 2.51, 95% CI 1.21–5.24; *p* = 0.014). After adjustment, breed, sex, age, current sickness, vaccination, and suckling status were not significant.

## 4. Discussion

Although Kabale, Rubanda, and Isingiro districts have previously reported Rift Valley fever (RVF) outbreaks, this study differs fundamentally from earlier investigations conducted in the same region. Rather than relying on outbreak-driven, facility-based, or ecologically targeted sampling, we implemented a probability-based, household-level serosurvey using randomly selected geocoded locations across all three districts. This design allowed us to assess RVF exposure at the population level within a historically affected region, independent of active outbreaks or perceived high-risk subareas. Our central finding is the unexpectedly high burden of prior RVF seropositivity in both humans and livestock, despite the absence of a contemporaneous outbreak. Human seroprevalence was 11.5%, animal-level seroprevalence was 14.6%, and 42.5% of herds had at least one seropositive animal, with cattle exhibiting the strongest serologic signal at 33.8%. The detection of substantial exposure through representative sampling within a post-outbreak setting indicates sustained, likely endemic transmission rather than residual clustering from past outbreaks. These findings refine existing outbreak-based narratives and highlight cattle-centered transmission interfaces and targeted behavioral interventions as immediate, practical priorities for RVF control in southwestern Uganda. Cattle act as key amplification hosts for RVFV, and mosquito vectors preferentially feed on cattle compared with other livestock species, thereby enhancing viral amplification and spillover risk to humans [15].

The observed human seroprevalence of 11.5% is comparable to estimates reported during the 2016 Kabale outbreak, where seropositivity reached approximately 13% [8]. However, the epidemiological context differs substantially. Whereas earlier estimates were derived during or shortly after recognized outbreaks, our findings demonstrate similarly high exposure levels in a randomly selected population sampled outside an outbreak period. This suggests that human RVF exposure in southwestern Uganda persists beyond outbreak detection windows and may be systematically underestimated by outbreak-based surveillance alone. Consistent with prior studies in Uganda, Kenya, and Tanzania, RVF seropositivity was higher among males and among adults aged 18–49 years, likely reflecting occupational and behavioral exposures such as livestock handling, slaughtering, and herding [16,17,18]. This pattern likely reflects occupational and behavioral exposures such as livestock handling, slaughtering, and herding, activities that are predominantly undertaken by men in many East African settings. Although male sex remained independently associated with RVF seropositivity after multivariable adjustment, gendered division of labor in livestock production may contribute to residual confounding and partially explain the observed sex differences in exposure risk. While Ahmed et al. reported higher seroprevalence in older adults in Tanzania, attributed to cumulative exposure opportunities over time [14], our findings indicate that economically active age groups bear a substantial burden of exposure. This likely reflects increased occupational engagement in livestock handling, herding, and related activities, which may elevate risk through both direct animal contact and closer proximity to mosquito vectors in livestock-rearing environments. These patterns have important implications for workforce productivity and household-level risk.

Cattle ownership emerged as one of the strongest independent predictors of human RVF seropositivity. Individuals from cattle-owning households were more than twice as likely to be seropositive, linking cattle infection to human spillover. This association has been reported previously in Uganda and Tanzania [9,16], but our study extends this evidence by demonstrating the relationship within a probability-sampled household population rather than outbreak-identified cases. Behavioral factors such as consumption of raw meat and involvement in slaughtering activities further support the importance of direct zoonotic transmission. Although individuals working closely with livestock may appear to be at increased risk, proximity to animals does not necessarily translate into greater exposure to RVF vectors, as *Aedes* and *Culex* mosquitoes preferentially feed on livestock, particularly cattle, when present. Consequently, human RVF infection in these settings is more likely to result from direct contact with infectious animal tissues and fluids rather than increased mosquito exposure. These practices have been implicated in severe RVF outbreaks elsewhere in East Africa, including the 2006–2007 Kenya outbreak, where handling of infected animal tissues was a major risk factor [19]. Together, these findings indicate that prevention strategies should prioritize human–cattle interfaces, including safer slaughter practices, carcass handling, and targeted behavior change communication and prevention of the disease in cattle.

Although raw milk consumption has been identified as an important route of RVFV transmission in other settings [20,21], it was not significantly associated with seropositivity in this study. This likely reflects the low prevalence of raw milk consumption among participants, particularly in Kabale and Rubanda districts, where milk is commonly boiled prior to consumption. The limited representation of pastoral populations may therefore have reduced the power to detect this exposure.

Another significant finding of this study is the herd-level seroprevalence of 42.5%, indicating that nearly half of the sampled herds had evidence of RVF exposure. To our knowledge, this represents the first household-based herd-level estimate in southwestern Uganda demonstrating such widespread exposure outside of a recognized outbreak [21]. This finding suggests sustained viral circulation within livestock populations, with ongoing potential for human spillover. Herd-level seroprevalence varied geographically, with particularly high levels in Rubanda District (51.6%), compared with Kabale (36.4%). Similar geographic heterogeneity has been reported in Kenya and Tanzania, where local ecology, livestock movement, and husbandry practices influence RVF persistence [22,23]. The close correspondence between district-level patterns in human and herd seroprevalence supports the presence of shared drivers such as cattle density, grazing practices, and vector ecology.

At the animal level, overall seroprevalence was 14.6%, with cattle showing markedly higher seropositivity (33.8%) compared with goats (6.7%) and sheep (7.3%). This species-specific pattern is consistent with previous national and regional surveys in Uganda, which have consistently identified cattle as having the highest RVF seroprevalence [10]. However, the added value of this study lies in demonstrating that cattle-associated seropositivity is directly linked to elevated human exposure within the same randomly sampled households. Age-related patterns in livestock suggested higher seroprevalence among younger animals, potentially reflecting immunological naivety or recent transmission. Animals with a history of illness had higher odds of seropositivity, supporting evidence that RVF infections in livestock are frequently mild or subclinical and may go unrecognized in endemic settings [24]. These findings reinforce the notion that passive surveillance based on clinical signs alone is insufficient to capture RVF transmission dynamics.

The presence of cattle within herds was strongly associated with herd-level seropositivity, consistent with evidence identifying cattle as efficient amplifying hosts for RVF virus [25,26]. Larger herd size was also associated with higher seroprevalence, likely reflecting increased livestock density, communal grazing, and greater exposure to infected vectors [27,28]. Environmental and management factors further shaped herd-level risk. Increased mosquito activity was associated with higher seropositivity, reinforcing the central role of vectors in RVF transmission [29]. Additionally, mosquito activity was assessed through self-reported measures, which may not accurately reflect true vector abundance. Future studies incorporating detailed entomological assessments and targeting pastoral communities may better elucidate the relative contributions of milk-borne and vector-mediated transmission pathways.

Although flooding was not statistically associated with seroprevalence in this study, this likely reflects limitations of cross-sectional serological data, which capture cumulative exposure rather than recent environmental conditions. Carcass management practices emerged as a critical risk factor. Herds that failed to bury carcasses properly had substantially higher odds of seropositivity, and on-farm slaughter was also associated with increased risk. These findings highlight how inadequate biosecurity can sustain viral circulation within herds and elevate zoonotic risk for humans involved in slaughtering and butchering [24].

Although this study was not designed to assess the availability or coverage of RVF vaccination in southwestern Uganda, anecdotal reports suggest that limited vaccination may occur in some settings. However, it is well established that the Government of Uganda has not fully rolled out a national RVF vaccination program for livestock, particularly cattle. Our findings demonstrate that cattle are the principal drivers of RVF transmission at the human–animal interface in this region. Consequently, we strongly recommend prioritizing vaccination of livestock, especially cattle, as a key intervention to reduce RVF infection in animals and, ultimately, to decrease zoonotic spillover into human populations. Given the central role of cattle as efficient amplifying hosts, vaccination of cattle has the potential to be a game changer for reducing human spillover by interrupting viral amplification at the animal source [30,31].

The strong concordance between human, animal, and herd-level seropositivity underscores the interconnectedness of human, animal, and environmental health in RVF epidemiology. Integrated surveillance systems that link veterinary and public health data are essential for early detection and prevention of spillover events. This study has limitations. Its cross-sectional design precludes causal inference, and serology reflects cumulative exposure rather than recent infection. Geographic restriction to southwestern Uganda limits generalizability. However, the use of randomized geocoded sampling and simultaneous assessment of humans and livestock provides a robust and policy-relevant understanding of RVF epidemiology in this region.

In conclusion, this study demonstrates substantial, previously underappreciated RVF exposure in southwestern Uganda using a representative, post-outbreak design. By identifying cattle as the dominant driver of human infection and revealing high herd-level prevalence, these findings support a shift from outbreak-centric responses toward sustained, cattle-focused, One Health prevention strategies.

## Figures and Tables

**Table 1:** Demographic factors associated with human RVF seropositivity in southwestern Uganda.

Characteristic	Category	N = 766	RVF Seronegative n (%)	RVF Seropositive n (%)	*p*-Value
District					0.04
	Isingiro	383	330 (86.16%)	53 (13.84%)	
	Kabale	205	191 (93.17%)	14 (6.83%)	
	Rubanda	178	157 (88.20%)	21 (11.80%)	
Age Category					<0.001
	≥50 years (Older adults)	249	203 (81.53%)	46 (18.47%)	
	31–49 years (Middle-aged)	273	244 (89.38%)	29 (10.62%)	
	18–30 years (Young)	180	167 (92.78%)	13 (7.22%)	
	0–17 years (Pediatric)	64	64 (100.00%)	0 (0.00%)	
Gender					0.10
	Female	428	386 (90.19%)	42 (9.81%)	
	Male	338	292 (86.39%)	46 (13.61%)	
Occupation					0.002
	Animal Husbandry	47	35 (74.47%)	12 (25.53%)	
	Crop Farming	322	287 (89.13%)	35 (10.87%)	
	Mixed Farming	240	209 (87.08%)	31 (12.92%)	
	Other Occupations	88	79 (89.77%)	9 (10.23%)	
	Student	69	68 (98.55%)	1 (1.45%)	

**Table 2:** Multivariate logistic regression analysis of risk factors for RVF Seropositivity in humans (N = 766).

Characteristic	Category	Adjusted OR	95% CI	*p*-Value
District	Isingiro (ref)	–	–	0.028
	Kabale	0.44	0.22–0.81	
	Rubanda	0.79	0.43–1.38	
Gender	Female (ref)	–	–	0.041
	Male	1.64	1.02–2.66	
Keeps Livestock	No (ref)	–	–	0.024
	Yes	0.51	0.27–0.92	
Keeps Cattle	No (ref)	–	–	0.006
	Yes	2.33	1.27–4.36	
Mosquito Increase	No (ref)	–	–	
	Yes	1.40	0.88–2.26	0.200
RVF Reports in Village	Don’t know (ref)	–	–	0.120
	No	0.49	0.26–1.01	
	Yes	0.32	0.05–1.34	
Age Category	Older adults (≥50 Years)	–	–	0.014
	Middle-aged (31–49 years)	2.02	1.20–3.48	
	Young Adults (18–30 years)	2.37	1.04–5.14	
Eats Raw Meat	No (ref)	–	–	0.035
	Yes	6.11	1.16–27.80	
Currently Unwell	No (ref)	–	–	0.004
	Yes	4.45	1.66–11.30	

OR = odds ratio; CI = confidence interval; ref = reference category.

**Table 3:** Multivariable (adjusted) logistic regression of factors associated with herd-level RVF seropositivity (N = 480 herds).

Characteristic	Category	aOR	95% CI	*p*-Value
District	Isingiro (ref)			
	Kabale	0.72	0.40–1.28	
	Rubanda	1.39	0.79–2.45	0.100
Livestock type: cattle	No (ref)			
	Yes	6.48	4.10–10.40	<0.001
Herd size	Large (ref)			
	Medium	0.39	0.19–0.79	<0.001
	Small	0.23	0.11–0.46	<0.001
Carcass disposal method	Burial with soil cover (ref)			
	No burial	15.70	4.23–63.60	<0.001
	Other disposal methods	1.10	0.48–2.49	0.823
	On-farm slaughter without safe disposal	2.14	1.21–3.89	0.010
Vaccination (self-reported)	No (ref)			
	Yes	1.63	0.89–2.97	0.110
Mosquito increase reported	No (ref)			
	Yes	1.75	1.13–2.73	0.012

**Table 4:** Multivariable logistic regression of animal-level RVF seropositivity (N = 2383).

Characteristic	Category	aOR	95% CI	*p*-Value
Species	Goat (ref)			
	Cattle	6.73	4.96–9.14	<0.001
	Sheep	1.10	0.74–1.65	0.626
Breed	Indigenous (ref)			
	Crossbreed	1.08	0.78–1.49	0.637
	Exotic	1.53	0.54–4.36	0.424
Sex	Female (ref)			
	Male	0.95	0.67–1.34	0.759
Age	Adult (≥24 months) (ref)			
	Middle-aged (12–<24 months)	1.05	0.67–1.63	0.842
	Young (<12 months)	0.66	0.43–1.03	0.066
Suckling status	No (ref)			
	Yes	1.65	0.96–2.84	0.068
RVF vaccinated	No (ref)			
	Yes	0.94	0.48–1.84	0.856
Illness history	No (ref)			
	Yes	2.58	1.22–5.43	0.013
Currently sick	No (ref)			
	Yes	0.47	0.17–1.32	0.150

## Data Availability

The data supporting the findings of this study are available within the article and its Supplementary Materials. Additional data may be made available from the corresponding author upon reasonable request. Data access is subject to ethical approvals and data-sharing agreements, as this study is part of an ongoing research project.

## Supplementary Materials

The following supporting information can be downloaded at: https://www.mdpi.com/article/10.3390/pathogens15020224/s1, Table S1: Bivariate analysis of environmental, management, and zoonotic risk factors for Rift Valley fever seropositivity at individual and herd levels.

## Abbreviations

The following abbreviations are used in this manuscript:

Abbreviation	Definition
aOR	Adjusted odds ratio
CI	Confidence interval
C-ELISA	Competitive enzyme-linked immunosorbent assay
IQR	Interquartile range
MAAIF	Ministry of Agriculture, Animal Industry and Fisheries
MOH	Ministry of Health
OD	Optical density
ODNC	Optical density of negative control
ODPC	Optical density of positive control
REC	Research Ethics Committee
RVF	Rift Valley fever
RVFV	Rift Valley fever virus
SPEAR	Seroprevalence and Ecologic Factors Associated with RVF
UNCST	Uganda National Council for Science and Technology
UVRI	Uganda Virus Research Institute
WSU	Washington State University
