# Supplementary material for: A Cross-Sectional Study of Rift Valley Fever Exposure in Humans and Livestock in Southwestern Uganda Using a One Health Approach: Evidence of Elevated Seroprevalence Outside Recognized Outbreak Periods
Source: Pathogens. 2026 Feb 17;15(2):224. doi: 10.3390/pathogens15020224 (PMC12943168; doi:10.3390/pathogens15020224)
Supplement: Supplementary file 1 [file pathogens-15-00224-s001.zip › pathogens-4135244-supplementary.pdf]

Supplementary Table 1: Bivariate Analysis of Environmental, Management, and Zoonotic Risk Factors for RVF Seropositivity

		Case			
**Characteristic**	**N**	**Overall**, N = 766[1]	**Negative**[1]	**Positive**[1]	**p-value**[2]
**Keep Livestock**	766				>0.9
No		289	256 (89%)	33 (11%)	
Yes		477	422 (88%)	55 (12%)	
**Keep Cattle**	766				**0.019**
No		559	504 (90%)	55 (9.8%)	
Yes		207	174 (84%)	33 (16%)	
**Keep Sheep**	766				0.6
No		590	524 (89%)	66 (11%)	
Yes		176	154 (88%)	22 (12%)	
**Keep Goats**	766				0.6
No		414	364 (88%)	50 (12%)	
Yes		352	314 (89%)	38 (11%)	
**Livestock System**	395				0.3
Communal		100	90 (90%)	10 (10%)	
Free-range		106	90 (85%)	16 (15%)	
Paddocking		13	12 (92%)	1 (7.7%)	
Ranching		4	2 (50%)	2 (50%)	
Tethering		148	132 (89%)	16 (11%)	
Zero-Grazing		24	21 (88%)	3 (12%)	
**Livestock _systems**	395				0.4
Controlled/Confinement Systems		185	165 (89%)	20 (11%)	
Free-Range Systems		210	182 (87%)	28 (13%)	
**Livestock mixing**	477				0.5
No		165	148 (90%)	17 (10%)	

Characteristic	N	Case<br>Overall, N = 766[1]	Negative[1]	Positive[1]	p-value[2]
Yes		312	274 (88%)	38 (12%)	
**Introduction of New Livestock**	477				0.5
No		434	382 (88%)	52 (12%)	
Yes		43	40 (93%)	3 (7.0%)	
**Slaughter at farm**	477				0.3
No		438	385 (88%)	53 (12%)	
Yes		39	37 (95%)	2 (5.1%)	
**Ectoparasite Control**	477				0.8
No		266	236 (89%)	30 (11%)	
Yes		211	186 (88%)	25 (12%)	
**Illness history on farm**	477				0.3
No		423	372 (88%)	51 (12%)	
Yes		54	50 (93%)	4 (7.4%)	
**Management of sick animals**	477				0.9
Treat by Vet		170	151 (89%)	19 (11%)	
Treat it myself		307	271 (88%)	36 (12%)	
**Carcass management**	188				0.7
Bury		124	112 (90%)	12 (9.7%)	
Other ways		64	59 (92%)	5 (7.8%)	
**Management Help**	477				0.2
No		377	337 (89%)	40 (11%)	
Yes		100	85 (85%)	15 (15%)	
**Vaccinated against any disease**	477				0.5
No		381	339 (89%)	42 (11%)	
Yes		96	83 (86%)	13 (14%)	
**Flooding**	766				0.7
No		651	575 (88%)	76 (12%)	

		Case			
**Characteristic**	**N**	**Overall**, N = 766[1]	**Negative**[1]	**Positive**[1]	**p-value**[2]
Yes		115	103 (90%)	12 (10%)	
**Mosquito Increase**	766				0.2
No		383	345 (90%)	38 (9.9%)	
Yes		383	333 (87%)	50 (13%)	
**Rice farming**	766				>0.9
No		763	675 (88%)	88 (12%)	
Yes		3	3 (100%)	0 (0%)	
**Quarry near**	766				0.3
No		711	627 (88%)	84 (12%)	
Yes		55	51 (93%)	4 (7.3%)	
**Presence of wild animals**	766				0.5
No		708	625 (88%)	83 (12%)	
Yes		58	53 (91%)	5 (8.6%)	
**RVF cases previously reported in Village**	766				0.3
Don't Know		76	63 (83%)	13 (17%)	
No		662	589 (89%)	73 (11%)	
Yes		28	26 (93%)	2 (7.1%)	
**Home water source**	766				**0.020**
Other		30	30 (100%)	0 (0%)	
Rain water		49	40 (82%)	9 (18%)	
River/Lake/Dam/Swamp/Pond		329	286 (87%)	43 (13%)	
Tap/ Piped water		190	176 (93%)	14 (7.4%)	
Well/Borehole		168	146 (87%)	22 (13%)	
**Home Water Source Type**	766				0.2
Engineered sources		437	392 (90%)	45 (10%)	
Natural sources		329	286 (87%)	43 (13%)	

		Case			
**Characteristic**	**N**	**Overall**, N = 766[1]	**Negative**[1]	**Positive**[1]	**p-value**[2]
**Zoonotic Link**	766				**0.016**
No		591	532 (90%)	59 (10.0%)	
Yes		175	146 (83%)	29 (17%)	
**Slept outside Home**	766				0.6
No		708	628 (89%)	80 (11%)	
Yes		58	50 (86%)	8 (14%)	
**Contact with Livestock**	766				0.5
No		302	270 (89%)	32 (11%)	
Yes		464	408 (88%)	56 (12%)	
**Home Slaughter**	766				>0.9
No		755	668 (88%)	87 (12%)	
Yes		11	10 (91%)	1 (9.1%)	
**Drink un-boiled Milk**	766				0.6
No		713	630 (88%)	83 (12%)	
Yes		53	48 (91%)	5 (9.4%)	
**Eat raw meat**	766				0.3
No		756	670 (89%)	86 (11%)	
Yes		10	8 (80%)	2 (20%)	
**Eat wild meat**	766				0.4
No		738	655 (89%)	83 (11%)	
Yes		28	23 (82%)	5 (18%)	
**Seen mosquitoes in House**	766				0.5
No		239	209 (87%)	30 (13%)	
Yes		527	469 (89%)	58 (11%)	
**Unwell Previously**	766				>0.9
No		703	622 (88%)	81 (12%)	
Yes		63	56 (89%)	7 (11%)	

		Case			
**Characteristic**	**N**	**Overall**, N = 766[1]	**Negative**[1]	**Positive**[1]	**p-value**[2]
**Currently unwell**	766				0.2
No		743	660 (89%)	83 (11%)	
Yes		23	18 (78%)	5 (22%)	
**Heard RVF**	766				0.8
No		621	549 (88%)	72 (12%)	
Yes		145	129 (89%)	16 (11%)	

. Bivariate Analysis: Herd Prevalence and Associated Factors

		Herd Prevalence			
**Characteristic**	**N**	**Overall**, N = 480[1]	**Negative**[1]	**Positive**[1]	**p-value**[2]
**District**	480				**0.045**
Isingiro		249	148 (59.44%)	101 (40.56%)	
Kabale		107	68 (63.55%)	39 (36.45%)	
Rubanda		124	60 (48.39%)	64 (51.61%)	
**Gender of Owner herd**	480				**0.040**
Female		219	137 (62.56%)	82 (37.44%)	
Male		261	139 (53.26%)	122 (46.74%)	

Characteristic	N	Herd Prevalence<br>**Overall**, N = 480[1]	**Negative**[1]	**Positive**[1]	**p-value**[2]
**Household Size-Categorise**	480				0.8
Large		85	50 (58.82%)	35 (41.18%)	
Medium		227	133 (58.59%)	94 (41.41%)	
Small		168	93 (55.36%)	75 (44.64%)	
**Own Cattle**	480				**<0.001**
No		275	211 (76.73%)	64 (23.27%)	
Yes		205	65 (31.71%)	140 (68.29%)	
**Own Sheep**	480				**0.016**
No		307	189 (61.56%)	118 (38.44%)	
Yes		173	87 (50.29%)	86 (49.71%)	
**Own Goats**	480				**<0.001**
No		131	58 (44.27%)	73 (55.73%)	
Yes		349	218 (62.46%)	131 (37.54%)	
**Herd Size**	480				**<0.001**
Large		86	21 (24.42%)	65 (75.58%)	
Medium		149	87 (58.39%)	62 (41.61%)	
Small		245	168 (68.57%)	77 (31.43%)	
**Purpose of Livestock**	480				**<0.001**
Dairy		68	18 (26.47%)	50 (73.53%)	
Dual		323	221 (68.42%)	102 (31.58%)	
Meat		16	7 (43.75%)	9 (56.25%)	
Other		73	30 (41.10%)	43 (58.90%)	
**Production system**	480				0.11
Extensive		140	78 (55.71%)	62 (44.29%)	
Intensive		276	168 (60.87%)	108 (39.13%)	

		Herd Prevalence			
**Characteristic**	**N**	**Overall**, N = 480[1]	**Negative**[1]	**Positive**[1]	**p-value**[2]
Semi-intensive		64	30 (46.88%)	34 (53.12%)	
**Mixing of Livestock**	480				0.5
No		175	104 (59.43%)	71 (40.57%)	
Yes		305	172 (56.39%)	133 (43.61%)	
**Introduction of New Livestock**	480				0.13
No		439	257 (58.54%)	182 (41.46%)	
Yes		41	19 (46.34%)	22 (53.66%)	
**Slaughter at farm**	480				0.4
No		443	257 (58.01%)	186 (41.99%)	
Yes		37	19 (51.35%)	18 (48.65%)	
**Spray against Ticks and Mosquitoes**	480				**<0.001**
No		270	173 (64.07%)	97 (35.93%)	
Yes		210	103 (49.05%)	107 (50.95%)	
**Animal illness history**	480				0.5
No		429	249 (58.04%)	180 (41.96%)	
Yes		51	27 (52.94%)	24 (47.06%)	
**Management of Sick animals**	480				0.5
other ways		25	14 (56.00%)	11 (44.00%)	
Treat by Self		289	172 (59.52%)	117 (40.48%)	
Treatment by Veterinarian		166	90 (54.22%)	76 (45.78%)	
**Carcass Management**	480				0.069
Bury		113	71 (62.83%)	42 (37.17%)	
No		13	5 (38.46%)	8 (61.54%)	
Others		59	40 (67.80%)	19 (32.20%)	

Characteristic	N	**Overall**, N = 480[1]	**Negative**[1]	**Positive**[1]	**p-value**[2]
		Herd Prevalence			
Slaughter		295	160 (54.24%)	135 (45.76%)	
**Vaccinated**	480				**<0.001**
No		386	239 (61.92 %)	147 (38.08%)	
Yes		94	37 (39.36%)	57 (60.64%)	
**Flooding**	480				0.8
No		409	234 (57.21%)	175 (42.79%)	
Yes		71	42 (59.15%)	29 (40.85%)	
**Seen More Mosquitoes in the last 3 months than Usual**	480				**0.009**
No		252	159 (63.10%)	93 (36.90%)	
Yes		228	117 (51.32%)	111 (48.68%)	
**Rice farming**	480				0.2
No		478	276 (57.74%)	202 (42.26%)	
Yes		2	0 (0.00%)	2 (100.00%)	
**Presence of quarry**	480				0.089
No		448	253 (56.47%)	195 (43.53%)	
Yes		32	23 (71.88%)	9 (28.12%)	
**Seen Wild animals near home**	480				0.7
No		443	256 (57.79%)	187 (42.21%)	
Yes		37	20 (54.05%)	17 (45.95%)	
**RVF Reports**	480				0.15
No		461	262 (56.83%)	199 (43.17%)	
Yes		19	14 (73.68%)	5 (26.32%)	
**Water Source**	480				0.7
Engineered Source		271	158 (58.30%)	113 (41.70%)	

Herd Prevalence					
**Characteristic**	**N**	**Overall**, N = 480[1]	**Negative**[1]	**Positive**[1]	**p-value**[2]
Natural Source		209	118 (56.46%)	91 (43.54%)	

[1]n (%)

[2]Pearson's Chi-squared test; Fisher's exact test
